# Burden and predictors of chronic obstructive pulmonary disease occurrence and severity among an occupational cohort of United States Department of Energy former workers

**DOI:** 10.1371/journal.pone.0322815

**Published:** 2025-05-06

**Authors:** Sara C. Howard, Louis Rocconi, Agricola Odoi

**Affiliations:** 1 Department of Biomedical and Diagnostic Sciences, The University of Tennessee, Knoxville, Tennessee, United States of America; 2 Epidemiology and Exposure Science, Oak Ridge Associated Universities, Oak Ridge, Tennessee, United States of America; 3 Department of Educational Leadership and Policy Studies, The University of Tennessee, Knoxville, Tennessee, United States of America; University of Oxford, UNITED KINGDOM OF GREAT BRITAIN AND NORTHERN IRELAND

## Abstract

**Background:**

Chronic Obstructive Pulmonary Disease (COPD) is a chronic inflammatory lung disease that reduces lung function and primarily affects older adults. Evidence suggests that occupational exposures like diesel exhaust, cadmium, welding fumes, and silica increase the risk of COPD. Some United States Department of Energy (DOE) workers may be exposed to these noxious substances as they execute their job responsibilities. Assessment of the burden of COPD among these workers and identification of the potential associations between the condition and the above occupational exposures is important for guiding screening, prevention, and control programs. Therefore, the objectives of this study are to: (a) estimate the burden of COPD among former workers of the DOE in the United States and (b) investigate the association between occupational exposures and COPD occurrence and severity among these workers while controlling for environmental, behavioral, and socio-demographic factors.

**Methods:**

Retrospective data containing health screening records of former DOE workers, covering the time period 2006–2019, were obtained from the National Supplemental Screening Program. Multivariate imputation by chained equation was used to impute missing values. Binary and multinomial logistic regression models were used to investigate predictors of COPD occurrence and severity, respectively.

**Results:**

Of the 17,376 participants included in the study, 20.8% had COPD. History of asthma, age at exam, body mass index, and smoking were significant predictors of both COPD occurrence and severity. Individuals exposed to silica had higher odds of COPD compared to those that were not exposed to silica. Similarly, diesel exhaust exposure was significantly associated with risk of more severe COPD.

**Conclusions:**

The findings of this study demonstrate the importance of considering occupational experience in the assessment of both COPD occurrence and severity. This information may be important for occupational screening programs as well as aiding in identifying modifiable risk factors to guide prevention and control efforts.

## Background

Chronic Obstructive Pulmonary Disease (COPD) is an inflammatory disease of the lungs, which obstructs airflow and reduces lung function [1]. Although the prevalence of the condition in the United States is only 6.2% [2], its annual economic burden is high and results in $32 billion in direct costs and $20.4 billion in indirect costs [3]. The prevalence of COPD increases with age, and hence older adults experience a higher burden of the condition. In the United States, approximately 10.6% of adults 55–64 years old and 12.8% of those ≥65 years old suffer from the condition [2]. Thus, COPD represents a significant challenge during aging. Evidence suggests that occupational exposures to substances such as cadmium [4], diesel exhaust [5], welding fumes [6], and silica [7] increase the risk of COPD. Approximately 31% (95% CI: 18–43%) of the COPD cases among non-smokers and 14% (95% CI: 10–18%) of cases among smokers are attributed to occupational exposures [8]. Historically, the United States Department of Energy (DOE) workers have been tasked with activities such as energy production, weapons design and production, and environmental cleanup, which may have resulted in occupational exposures of noxious substances [9]. As a result, the DOE instituted the Former Worker Medical Screening Program (FWP), which is comprised of six individual surveillance programs, for screening and early identification of diseases, such as COPD [9].

Understanding the association between occupational exposures and risk of COPD while controlling for environmental (e.g., air quality) [3], socio-demographic, and behavioral (e.g., smoking) factors [10], among aging DOE former workers is important for guiding disease prevention/control and reduction of healthcare costs associated with the condition. Therefore, the objectives of this study were to: (a) estimate the burden of COPD among former workers of the DOE in the United States and (b) investigate occupational predictors of COPD among these former workers while controlling for environmental, behavioral, and socio-demographic factors.

## Methods

### Ethics approval

This study was reviewed and approved by the United States Central Department of Energy Institutional Review Board (DOE ID: DOE000645). The need for consent was waived by the Institutional Review Board. No minors were included in the study since it involved only DOE former workers.

### Study population

This retrospective study used data collected as part of the National Supplemental Screening Program (NSSP), which is one of the six surveillance programs under the Former Worker Program (FWP), and is the only national program for non-construction former workers. These data were accessed for research purposes on August 26, 2021 in an anonymized form to ensure that the investigators could not identify participants during or after data collection. The NSSP provides free, voluntary medical screening to DOE former workers, contractors, and subcontractors across all 50 states of the United States. Repeated screening exams are offered to participants every three years [9,11]. Only participants who completed an initial screening exam that included a spirometry test from January 1, 2006, to December 31, 2019, were included in this study (n = 17,960). Individuals who reported a history of restrictive lung disease including silicosis, asbestosis, and chronic beryllium disease at the time of the initial exam (n = 584) were excluded to avoid false positives since obstructive lung function patterns, similar to COPD, have been reported among individuals with restrictive lung diseases [12]. In some instances, participants had some aspects of their initial screening exams repeated (n = 2,230). When this occurred, the most recent exam results were used. The final study cohort included 17,376 participants.

### Data sources, description, preparation, and management

#### Chronic obstructive pulmonary disease (COPD) data.

Spirometry measurements, which assess lung function, were used to identify COPD based on the **G**lobal Initiative for Chronic **O**bstructive **L**ung **D**isease (GOLD) criterion [3]. Using this criterion, participants with COPD were identified as those with forced expiratory volume in one second (FEV_1_) to forced vital capacity (FVC) ratio less than 0.7 (i.e., FEV_1_/FVC < 0.7). The severity of the condition was also graded using the GOLD criterion as mild (FEV_1_ ≥ 80% of predicted), moderate (FEV_1_ 50–79% of predicted), severe (FEV_1_ 30–49% of predicted), and very severe (FEV_1_ < 30% of predicted) [3,13]. For investigation of predictors of COPD, two outcome variables were used: COPD occurrence (yes/no) and COPD severity (very severe, severe, moderate, mild). Due to the small number of NSSP participants with very severe COPD, this category was combined with the severe category to avoid the small number problem resulting in unreliable/unstable estimates during statistical analysis.

#### Occupational exposures data.

Occupational exposures to cadmium, diesel exhaust, welding fumes, and silica were assessed using the exposure history available in the NSSP at the time of initial screening exams. Participants were asked if they were exposed to each of the substances as a normal part of their job duties. Response options were dichotomous (yes/no). Job title for each participant was provided by the NSSP as one of twelve standardized job titles: administrative support, biohazard, crafts, field professionals, guests, in-house professionals, line operators, management, security/fire protection, service, technical support, and unknown. For participants who had more than one job title, the longest held title was recorded as the job title. The twelve standardized job titles were classified into three occupational exposure categories (low, medium, and high) based on potential level of occupational hazards as described in a previous DOE surveillance program which used the same standardized job titles [14]. The low exposure category included individuals involved in administrative support, management, and in-house professionals while the medium exposure category included field-professionals, biohazard, security/fire protection, and guests. Line operators, crafts, service, and technical support job titles were categorized in the high exposure risk category. The DOE facility where the worker spent most of their career was recorded as their primary site. The facilities were categorized as weapons production, uranium processing, or science and laboratory facilities. Hire and termination dates were provided as month/year and were used to calculate duration of employment, which was then categorized into 5-year time periods.

#### Smoking data.

Data on lifetime smoking habits were captured at the time of the initial exams. Participants were categorized as ever smokers or never smokers based on whether they ever smoked more than 20 packs of cigarettes or if they were smokers at the time of the screening.

#### Air quality data.

Air quality data were downloaded from the Environmental Protection Agency (EPA) website [15]. Data on mean concentration of air pollutants were used to calculate the EPA’s air quality index (AQI) per zip code per year. To calculate AQI, the mean concentrations for ozone, carbon monoxide, sulfur dioxide, nitrogen dioxide, and particulate matter (PM_2.5_ and PM_10_) were calculated per zip code. Using EPA’s technical basis document for air quality, the mean values were converted to AQI values and categorized as good (AQI: < 51), moderate (AQI: 51–100), unhealthy for sensitive groups (AQI: 101–150), unhealthy (AQI: 151–200), very unhealthy (AQI: 201–300), and hazardous (AQI: 301–500) [16].

Study participants were matched to AQI based on their zip code of residence and year of their initial exam. If a participant’s zip code did not contain air quality data, then AQI of the closest zip code was assigned. To find the nearest zip code, the zip codes were first converted to zip code tabulation areas (ZCTAs) using the crosswalk developed by USA Mapper [17] and then matched to the US Census Bureau’s TIGER/Line shapefiles [18]. The centroid of the ZCTAs were then used to calculate the distance to the nearest ZCTA.

### Statistical analyses

#### Descriptive analysis.

All statistical analyses were performed in SAS 9.4 [19] and R version 4.0.3 [20] using RStudio version 2021.09.2 interface [21]. Frequencies and 95% confidence intervals were computed for categorical variables.

#### Multiple imputation.

Due to the high proportion of missing values for race (22.3%) as well as smaller proportions of missing data in potential predictors of COPD occurrence and severity, multivariate imputation by chained equation (MICE) was performed on the dataset using the MICE package [22] in RStudio version 2021.09.2 [21] interface of R [20]. The MICE procedure used logistic regression for binary variables and multinomial logistic regression for categorical variables with more than two categories [22]. Since the outcome variable of interest should be included in the multiple imputation model [23], multiple imputed (or MICE) datasets were created separately for the two outcome variables of interest: COPD occurrence (yes/no) and COPD severity (very severe, severe, moderate, mild). Multiple imputation by chained equations was conducted for all potential predictors of COPD occurrence and COPD severity separately. For both the COPD occurrence and COPD severity imputations, the number of imputed datasets was 20 and the maximum number of iterations for the imputation was 30. The choice of the number of imputations was based on the fraction of missing data and on limiting the decrease in power as outlined by Graham et al. [24]. The results of the statistical models performed on the multiple imputed datasets were pooled in accordance with Rubin’s rules for pooling, which accounts for the additional variability produced by multiple imputation [25].

#### Identification of highly correlated potential predictors.

To avoid multicollinearity, pairwise Spearman’s rank correlation coefficients were computed to identify highly correlated (|r|>0.70) potential predictors. If a pair of variables was highly correlated, only one of them was included for assessment in the multivariable models. However, none of the variables were highly correlated (Table 1).

**Table 1:** Spearman rank correlation coefficients for potential predictors of chronic obstructive pulmonary disease among the National Supplemental Screening Program participants, 2006-2019.

	Body Mass Index	Air Quality Index	Sex	Race	Smoking Status	DOE Site	Asthma	Cadmium	Diesel Exhaust	Silica	Welding Fumes	Exposure Potential	Work Duration	Age
Body Mass Index	1.000	−0.016	0.007	0.090	0.036	0.097	0.062	0.047	0.082	0.056	0.083	0.074	0.035	−0.073
Sex	0.007	−0.011	1.000	0.014	0.000	0.013	0.000	0.006	0.006	0.002	0.002	0.004	0.002	−0.011
Race	0.090	−0.049	0.014	1.000	−0.069	0.116	0.023	0.013	0.074	−0.001	0.027	0.081	0.045	−0.108
Smoking Status	0.036	0.066	0.000	−0.069	1.000	0.077	−0.021	0.068	0.066	0.066	0.116	0.135	0.094	0.179
DOE Site	0.097	0.036	0.013	0.116	0.077	1.000	0.008	0.111	0.132	0.081	0.126	0.165	0.143	0.063
Asthma	0.062	0.009	0.000	0.023	−0.021	0.008	1.000	0.002	0.013	0.004	0.003	−0.023	−0.018	−0.053
Cadmium	0.047	0.084	0.006	0.013	0.068	0.111	0.002	1.000	0.230	0.370	0.328	0.166	0.197	0.008
Diesel Exhaust	0.082	−0.011	0.006	0.074	0.066	0.132	0.013	0.230	1.000	0.303	0.361	0.132	0.168	−0.040
Silica	0.056	0.046	0.002	−0.001	0.066	0.081	0.004	0.370	0.303	1.000	0.364	0.133	0.160	−0.029
Welding Fumes	0.083	0.050	0.002	0.027	0.116	0.126	0.003	0.328	0.361	0.364	1.000	0.193	0.218	0.008
Exposure Potential	0.074	0.103	0.004	0.081	0.135	0.165	−0.023	0.166	0.132	0.133	0.193	1.000	0.135	0.004
Work Duration	0.035	0.005	0.002	0.045	0.094	0.143	−0.018	0.197	0.168	0.160	0.218	0.135	1.000	0.239
Age	−0.073	−0.034	−0.011	−0.108	0.179	0.063	−0.053	0.008	−0.040	−0.029	0.008	0.004	0.239	1.000

#### Predictors of COPD occurrence.

To identify potential predictors of COPD occurrence (yes/no), logistic regression models were fit using RStudio version 2021.09.2 [21] interface of R [20] for multiple imputation data. Initially, univariable logistic regression models were fit, and the independent variables with a p<0.20 were considered for the multivariable logistic regression model. The reference categories for the logistic regression models were chosen with a priori knowledge of the data and the possible direction of association with COPD. Backwards elimination was used to build the multivariable logistic model using an alpha of 0.05. An independent variable was considered a confounder if its removal from the model resulted in at least a 20% change in the regression coefficients of any of the variables still in the model [26]. Goodness of fit of the final model was assessed using the Hosmer-Lemeshow test [27].

#### Predictors of COPD severity.

For COPD severity, only participants with COPD were included in the model. Due to the small number of participants with very severe COPD, the severity variable was re-coded from a 4-categeory variable (very severe, severe, moderate, mild) to a 3-category (severe, moderate, mild) variable by combining the very severe and severe categories. Multinomial logistic regression models were used to identify potential predictors of COPD severity in RStudio 2021.09.2 interface [21] of R [20] for analyses with MICE data. As with the ordinary logistic regression models, a relaxed critical value of p<0.20 was used to determine which independent variables from the univariable multinomial logistic regression models would be included in the multivariable multinomial logistic regression model. Variables were removed from the multivariable multinomial logistic regression model using backwards elimination when the p>0.05. An independent variable was considered a confounder if its removal from the model resulted in at least a 20% change in the regression coefficients of any of the variables still in the model [26]. The exponentiated regression coefficients of the multinomial logistic regression models are Relative Risk Ratios (RRR) which are similar to odds ratios in interpretation [28]. Thus, the RRR is a ratio of the relative risks produced by the multinomial model for each level of the outcome compared to the baseline outcome level [26,28]. The reference categories for each variable were selected based on a priori knowledge of the data and the hypothesized/plausible association with COPD. For example, advanced age tends to be associated with higher risk of COPD [3]. Therefore, the reference category for the age variable would be the lowest age category.

## Results

### Descriptive statistics

The study included 17,376 DOE former workers who participated in the NSSP from 2006 through 2019. Based on the GOLD criteria, 20.8% of the study participants had COPD (Table 2). A total of 43.0% of the COPD cases were classified as mild, 42.8% as moderate, and 14.2% as severe or very severe. The study population was predominantly non-black (88.1%) and male (74.7%). There was a fairly even spread across age categories with 17.9% less than 55 years old, 12.0% 55–59 years old, 16.5% 60–64 years old, 17.3% 65–69 years old, 14.3% 70–74 years old, and 21.5% ≥ 75 years old. The majority of the study cohort were overweight (39.9%) or obese (39.8%) while only 19.9% had normal weight and an additional 0.5% were underweight. Less than a half (43.5%) were ever smokers, and 13.2% reported a history of asthma. The majority (85.7%) of the study population lived in areas where the air quality index (AQI) was considered good whereas 14.3% lived in areas where the AQI was moderate or unhealthy for sensitive groups.

**Table 2:** Summary statistics of the characteristics of National Supplemental Screening Program participants at initial examination, 2006–2019.

Characteristics	Frequency	Percentage	95% Confidence Interval
**Chronic Obstructive Pulmonary Disease Occurrence (COPD)**	**n = 17,376**		
No	13,755	79.2	78.6, 79.8
Yes	3,621	20.8	20.2, 21.44
**Chronic Obstructive Pulmonary Disease Severity**	**n = 3,621**		
Mild	1,557	43.0	41.4, 44.6
Moderate	1,549	42.8	41.2, 44.4
Severe or Very Severe	515	14.2	13.1, 15.4
**Race**	**n = 13,515**		
Black	1,612	11.9	11.4, 12.5
Non-Black	11,903	88.1	87.5, 66.6
**Sex**	**n = 17,376**		
Male	12,978	74.7	74.0, 75.3
Female	4,398	25.3	24.7, 26.0
**Age Categories**	**n = 17,376**		
< 55 years old	3,112	17.9	17.3, 18.5
55-59 years old	2,085	12.0	11.5, 12.5
60-64 years old	2,871	16.5	16.0, 17.1
65-69 years old	3,010	17.3	16.8, 17.9
70-74 years old	2,562	14.7	14.2, 15.3
≥ 75 years old	3,736	21.5	20.9, 22.1
**Body Mass Index (BMI) Category**	**n = 17,333**		
Underweight	82	0.47	0.37, 0.59
Normal	3,442	19.9	19.3, 20.5
Overweight	6,910	39.9	39.1, 40.6
Obese & Extremely Obese	6,899	39.8	39.1, 40.5
**Smoking Status**	**n = 17,376**		
Non-Smoker	9,825	56.5	55.8, 57.3
Ever Smoker	7,551	43.5	42.7, 44.2
**History of Asthma**	**n = 16,932**		
No	14,697	86.8	86.3, 87.3
Yes	2,235	13.2	12.7, 13.71
**Air Quality Index (AQI) Category**	**n = 17,338**		
Good	14,862	85.7	85.2, 86.2
Moderate or Unhealthy for Sensitive Groups	2,476	14.3	13.8, 14.8
**Department of Energy Site Category**	**n = 17,376**		
Science and Laboratory Facilities	3,600	20.7	20.1, 21.3
Uranium Processing Facilities	1,125	6.5	6.1, 6.8
Weapons Production Facilities	12,651	72.8	72.2, 73.5
**Working Duration**	**n = 17,376**		
< 5 years	5,594	32.2	31.5, 32.9
5-9 years	2,879	16.6	16.0, 17.1
10-14 years	1,998	11.5	11.0, 12.0
15-19 years	1,547	8.9	8.5, 9.3
20-24 years	1,368	7.9	7.5, 8.3
25-29 years	1,212	7.0	6.6, 7.4
≥ 30 years	2,778	16.0	15.4, 16.5
**Exposure Potential**	**n = 17,140**		
Low	4,568	26.7	26.0, 27.3
Medium	4,946	28.9	28.2, 29.5
High	7,626	44.5	43.8, 45.2
**Occupational Exposure to Cadmium**	**n = 17,343**		
No	9,188	53.0	64.5, 65.9
Yes	8,148	47.0	34.1, 35.5
**Occupational Exposure to Welding Fumes**	**n = 17,336**		
No	9,188	53.0	52.3, 53.7
Yes	8,148	47.0	46.3, 47.7
**Occupational Exposure to Silica**	**n = 17,340**		
No	11,308	65.2	52.3, 53.7
Yes	6,032	34.8	46.3, 47.7
**Occupational Exposure to Diesel Exhaust**	**n = 17,352**		
No	12,313	71.0	70.3, 71.6
Yes	5,039	29.0	28.4, 29.7

Most of the study population were employed at weapons production facilities (72.8%), followed by science and laboratory facilities (20.7%), and uranium processing facilities (6.5%). The majority were employed at a DOE facility for less than 15 years (<5 years: 32.2%, 5–9 years: 16.6%, 10–14 years: 11.5%) although 16.0% were employed for at least 30 years. Regarding potential for occupational exposures, 26.7% of the study participants were classified as having low potential for exposure, 28.9% were classified as medium potential, and 44.5% were classified as high potential. Welding fumes and cadmium were the most commonly reported occupational exposures at 47.0% of the cohort each, with silica at 34.8% and diesel exhaust at 29.0%.

### Predictors of COPD occurrence

Of the potential predictors investigated, only sex and AQI category did not have univariable associations (p > 0.2) with COPD occurrence (Table 3). However, based on the multivariable model, significant (p < 0.05) predictors of COPD occurrence were age category, BMI category, smoking, history of asthma, DOE facility, and occupational exposure to silica (Table 4). The odds of COPD were higher across all age categories compared to those < 55 years old, and the odds ratios steadily increased with each advancing age category, reaching its highest value of 3.184 (95% CI: 2.786, 3.638) among those ≥75 years old. Individuals who were underweight had higher odds of COPD (OR: 2.161; 95% CI: 1.355, 3.445) compared to normal weight individuals. However, those who were overweight (OR: 0.676; 95% CI: 0.612, 0.746) or obese (OR: 0.513: 95% CI: 0.463, 0.569) had lower odds of COPD compared to individuals with normal weight. The odds of COPD occurrence were 2.029 times (95% CI: 1.877, 2.193) higher among ever smokers than non-smokers. Individuals with a self-reported history of asthma had 2.259 times (95% CI: 2.035, 2.508) higher odds of COPD occurrence compared to those without history of asthma. Additionally, NSSP participants who were employed at uranium processing facilities (OR: 1.255; 95% CI: 1.057, 1.491) or weapons production facilities (OR: 1.173; 95% CI: 1.061, 1.296) had higher odds of COPD occurrence compared to participants employed at science and laboratory facilities. The Hosmer-Lemeshow goodness of fit test p-value was p = 0.0872 implying a good model fit.

**Table 3:** Unadjusted logistic regression model results investigating predictors of chronic obstructive pulmonary disease occurrence among the National Supplemental Screening Program participants, 2006–2019.

Characteristics	Odds Ratio	95% Confidence Interval	p-value	Overall p-value
**Race**				<0.001
Black	0.776	0.673, 0.894	<0.001	
Non-Black	*Reference*			
**Sex**				0.683
Female	0.983	0.903, 1.069	0.683	
Male	*Reference*			
**Age Categories**				<0.001
55-59 years old	1.295	1.010, 1.524	0.002	
60-64 years old	1.645	1.423, 1.902	<0.001	
65-69 years old	1.944	1.690, 2.238	<0.001	
70-74 years old	2.292	1.987, 2.642	<0.001	
≥ 75 years old	3.645	3.204, 4.146	<0.001	
< 55 years old	*Reference*			
**Body Mass Index (BMI) Categories**				<0.001
Underweight	2.21	1.421, 3.436	<0.001	
Overweight	0.724	0.658, 0.795	<0.001	
Obese & Extremely Obese	0.551	0.499, 0.608	<0.001	
Normal	*Reference*			
**Smoking Status**				<0.001
Ever Smoker	2.196	2.039, 2.366	<0.001	
Non-Smoker	*Reference*			
**History of Asthma**				<0.001
Yes	1.902	1.721, 2.101	<0.001	
No	*Reference*			
**Air Quality Index (AQI) Category**				0.412
Moderate or Unhealthy for Sensitive Groups	1.044	0.941, 1.159	0.412	
Good	*Reference*			
**Department of Energy Site Category**				<0.001
Uranium Processing Facilities	1.296	1.099, 1.526	0.002	
Weapons Production	1.26	1.145, 1.285	<0.001	
Science and Laboratory Facilities	*Reference*			
**Working Duration**				<0.001
5-9 years	1.028	0.918, 1.152	0.629	
10-14 years	1.128	0.994, 1.280	0.061	
15-19 years	1.076	0.935, 1.238	0.306	
20-24 years	1.012	0.872, 1.175	0.871	
25-29 years	1.295	1.117, 1.502	<0.001	
≥ 30 years	1.34	1.201, 1.495	<0.001	
< 5 years	*Reference*			
**Exposure Potential**				0.024
Medium	0.917	0.829, 1.014	0.091	
High	1.038	0.948, 1.136	0.417	
Low	*Reference*			
**Occupational Exposure to Cadmium**				0.063
Yes	1.075	0.996, 1.160	0.063	
No	*Reference*			
**Occupational Exposure to Welding Fumes**				0.009
Yes	1.103	1.025, 1.187	0.009	
No	*Reference*			
**Occupational Exposure to Silica**				<0.001
Yes	1.139	1.056, 1.229	<0.001	
No	*Reference*			
**Occupational Exposure to Diesel Exhaust**				0.090
Yes	1.072	0.989, 1.161	0.090	
No	*Reference*			

**Table 4:** Predictors of chronic obstructive pulmonary disease occurrence among National Supplemental Screening Program participants, 2006-2019.

Characteristics	Odds Ratio	95% Confidence Interval	p-value	Overall p-value
**Age Categories**				<0.001
55-59 years old	1.249	1.057, 1.476	0.009	
60-64 years old	1.552	1.337, 1.800	<0.001	
65-69 years old	1.797	1.555, 2.076	<0.001	
70-74 years old	2.085	1.800, 2.415	<0.001	
≥ 75 years old	3.184	2.786, 3.638	<0.001	
< 55 years old	*Reference*			
**Body Mass Index (BMI) Categories**				<0.001
Underweight	2.161	1.355, 3.445	0.001	
Overweight	0.676	0.612, 0.746	<0.001	
Obese & Extremely Obese	0.513	0.463, 0.569	<0.001	
Normal	*Reference*			
**Smoking Status**				<0.001
Ever Smoker	2.029	1.877, 2.193	<0.001	
Non-Smoker	*Reference*			
**History of Asthma**				<0.001
Yes	2.259	2.035, 2.508	<0.001	
No	*Reference*			
**Department of Energy Site Category**				0.003
Uranium Processing Facilities	1.255	1.057, 1.491	0.009	
Weapons Production	1.173	1.061, 1.296	0.002	
Science and Laboratory Facilities	*Reference*			
**Occupational Exposure to Silica**				<0.001
Yes	1.157	1.068, 1.253	<0.001	
No	*Reference*			

### Predictors of COPD severity

With the exception of sex, race, and work duration, all assessed potential predictors had univariable associations (p < 0.2) with COPD severity (Table 5). The final multivariable multinomial logistic model included age category, BMI category, smoking, history of asthma, DOE facility category, and occupational exposure to diesel exhaust as significant (p < 0.05) predictors of COPD severity (Table 6). While most of the predictors had similar relative risk ratios (RRR) for moderate disease and severe or very severe disease, smoking status and history of asthma had higher RRRs for severe or very severe disease compared the RRRs for moderate disease. For instance, the risk of moderate COPD among ever smokers was 1.606 (95% CI: 1.385, 1.863) times higher than the risk in non-smokers, but the risk of severe or very severe COPD was 2.720 (95% CI: 2.163, 3.420) times higher among ever smokers than non-smokers. A similar difference in the association was observed among participants with asthma. The risk for moderate COPD among those with asthma was 1.991 (95% CI: 1.638, 2.420) times higher than among those without asthma. However, the risk of severe or very severe COPD among individuals with asthma was 3.362 (95% CI: 2.607, 4.336) times higher than that among those without asthma. Conversely, the overweight RRR was 1.186 (95% CI: 0.989, 1.423) for moderate COPD and 0.757 (95% CI: 0.584, 0.982) for severe or very severe COPD while obese had similar RRRs across both moderate (RRR: 1.751 95% CI: 1.442, 2.128) and severe or very COPD (RRR: 1.239, 95% CI: 0.947, 1.620). The underweight RRR was higher for both moderate (RRR: 2.933, 95% CI: 1.179, 7.297) and severe or very severe COPD (RRR: 5.749, 95% CI: 2.203, 15.008) compared to normal BMI.

**Table 5:** Unadjusted multinomial regression model results investigating predictors of chronic obstructive pulmonary disease severity among the National Supplemental Screening Program participants, 2006-2019.

	Moderate			Severe or Very Severe			Overall
**Characteristics**	**RRR**	**95% CI**	**p-value**	**RRR**	**95% CI**	**p-value**	**P-value**
**Race**							0.471
Black	0.811	0.620, 1.062	0.127	1.038	0.708, 1.521	0.848	
Non-Black	*Reference*						
**Sex**							0.6710
Female	1.060	0.901, 1.246	0.495	0.968	0.767, 1.221	0.782	
Male	*Reference*						
**Age Categories**							<0.001
55-59 years old	1.427	1.037, 1.964	0.029	1.316	0.739, 2.342	0.351	
60-64 years old	1.352	1.017, 1.798	0.038	2.028	1.253, 3.283	0.004	
65-69 years old	1.58	1.198, 2.085	0.001	2.884	1.822, 4.566	<0.001	
70-74 years old	1.267	0.958, 1.675	0.097	2.75	1.740, 4.345	<0.001	
≥ 75 years old	1.557	1.214, 1.998	<0.001	2.789	1.815, 4.296	<0.001	
< 55 years old	*Reference*						
**Body Mass Index (BMI) Categories**							<0.001
Underweight	3.049	1.240, 7.496	0.015	6.243	2.471, 15.772	<0.001	
Overweight	1.213	1.015, 1.450	0.034	0.799	0.621, 1.027	0.080	
Obese & Extremal Obese	1.837	1.521, 2.220	<0.001	1.361	1.053, 1.758	0.018	
Normal	*Reference*						
**Smoking Status**							<0.001
Ever Smoker	1.651	1.431, 1.905	<0.001	2.854	2.288, 3.560	<0.001	
Non-Smoker	*Reference*						
**History of Asthma**							<0.001
Yes	1.905	1.574, 2.306	<0.001	2.92	2.282, 3.737	<0.001	
No	*Reference*						
**Air Quality Index (AQI) Category**							0.0907
Moderate or Unhealthy for Sensitive Groups	1.239	1.014, 1.514	0.036	1.176	0.887, 1.559	0.259	
Good	*Reference*						
							
**Department of Energy Site Category**							<0.001
Uranium Processing Facilities	1.744	1.264, 2.407	<0.001	2.345	1.507, 3.650	<0.001	
Weapons Production	1.568	1.303, 1.887	<0.001	1.776	1.338, 2.356	<0.001	
Science and Laboratory Facilities	*Reference*						
**Working Duration**							0.279
5-9 years	1.11	0.893, 1.380	0.347	1.102	0.799, 1.518	0.554	
10-14 years	1.125	0.880, 1.439	0.347	1.617	1.163, 2.247	0.004	
15-19 years	1.09	0.866, 1.426	0.527	1.068	0.718, 1.589	0.745	
20-24 years	1.131	0.834, 1.504	0.399	0.962	0.620, 1.494	0.864	
25-29 years	1.168	0.850 1.551	0.283	1.459	0.989, 2.152	0.057	
≥ 30 years	1.045	0.880, 1.287	0.680	1.326	0.990, 1.776	0.058	
< 5 years	*Reference*						
**Exposure Potential**							<0.001
Medium	1.144	0.944, 1.386	0.171	0.88	0.662, 1.170	0.380	
High	1.333	1.121, 1.585	0.001	1.399	1.098, 1.782	0.007	
Low	*Reference*						
**Occupational Exposure to Cadmium**							0.130
Yes	1.126	0.972, 1.303	0.113	1.206	0.982, 1.482	0.074	
No	*Reference*						
**Occupational Exposure to Welding Fumes**							<0.001
Yes	1.193	1.036, 1.374	0.014	1.439	1.177, 1.759	<0.001	
No	*Reference*						
**Occupational Exposure to Silica**							0.008
Yes	1.102	0.952, 1.276	0.194	1.373	1.120, 1.682	0.002	
No	*Reference*						
**Occupational Exposure to Diesel Exhaust**							<0.001
Yes	1.348	1.155, 1.574	<0.001	1.388	1.119, 1.721	0.003	
No	*Reference*						

**Table 6:** Predictors of chronic obstructive pulmonary disease severity among National Supplemental Screening Program participants, 2006-2019.

	Moderate			Severe or Very Severe			Overall
**Characteristics**	**RRR**	**95% CI**	**p-value**	**RRR**	**95% CI**	**p-value**	**p-value**
**Age Categories**							<0.0001
55-59 years old	1.217	0.875, 1.692	0.2441	1.004	0.554, 1.818	0.9899	
60-64 years old	1.132	0.842, 1.520	0.4115	1.539	0.936, 2.532	0.0896	
65-69 years old	1.341	1.007, 1.787	0.0448	2.228	1.386, 3.582	0.0009	
70-74 years old	1.124	0.841, 1.501	0.4307	2.256	1.405, 3.623	0.0008	
≥ 75 years old	1.465	1.131, 1.898	0.0039	2.402	1.540, 3.746	0.0001	
< 55 years old	*Reference*						
**Body Mass Index (BMI) Categories**							<0.0001
Underweight	2.933	1.179, 7.297	0.0207	5.749	2.203, 15.008	0.0004	
Overweight	1.186	0.989, 1.423	0.0655	0.757	0.584, 0.982	0.0362	
Obese & Extremely Obese	1.751	1.442, 2.128	<0.0001	1.239	0.947, 1.620	0.1177	
Normal	*Reference*						
**Smoking Status**							<0.0001
Ever Smoker	1.606	1.385, 1.863	<0.0001	2.720	2.163, 3.420	<0.0001	
Non-Smoker	*Reference*						
**History of Asthma**							<0.0001
Yes	1.991	1.638, 2.420	<0.0001	3.362	2.607, 4.336	<0.0001	
No	*Reference*						
**Department of Energy (DOE) Site Category**							0.0002
Uranium Processing Facilities	1.584	1.139, 2.204	0.0063	2.185	1.379, 3.461	0.0009	
Weapons Production	1.432	1.183, 1.733	0.0002	1.578	1.177, 2.118	0.0023	
Science and Laboratory Facilities	*Reference*						
**Occupational Exposure to Diesel Exhaust**							0.0126
Yes	1.243	1.060, 1.460	0.0075	1.273	1.016, 1.594	0.0357	
No	*Reference*						

## Discussion

This study investigated predictors of COPD occurrence and severity among an occupational cohort of DOE former workers in the United States. The findings from this study will be useful for improving screening guidelines for occupational screening programs such as the National Supplemental Screening Program (NSSP).

The results of this study show that predictors of COPD occurrence and severity are quite similar in the NSSP population. Similar to reports from other studies [1–3,10,29–31] this study found significant associations between COPD and both smoking and age. Moreover, consistent with findings from several other studies [30,32–34], this study found that, compared to normal BMI, low BMI (underweight) was associated with higher odds of COPD whereas high BMI (overweight/obese) was associated with lower odds of COPD. The associations between low BMI (vs normal BMI) and higher risks of both moderate and severe COPD, observed in this study, is consistent with reports from other studies [35,36]. However, this should be interpreted with caution due to potential reverse causation bias since severe COPD can lead to weight loss resulting in low BMI and therefore complicating interpretation [37]. Moreover, the cross-sectional nature of the study does not allow for assessment of temporality of events or causal inference [26]. Therefore, further investigation is required to fully understand the association between BMI and COPD severity. It is worth noting that, in this study, there was no evidence of association between high BMI (overweight/obese) and lower risk of moderate or severe COPD, which has been reported by other studies [36].

The finding that history of asthma is a predictor of COPD occurrence and severity is consistent with results from a few other studies [38,39] although the underlying biological mechanism of the observed association between asthma and COPD is not well understood. Some studies have suggested that history of asthma, particularly in childhood, may impact lung function over a lifetime possibly leading to COPD and possibly declining lung function, which could be indicative of more severe COPD [39]. In recent years, asthma-COPD overlap syndrome, in which both asthma and COPD occur simultaneously in the same individual, has begun to be recognized as a distinct condition that may have different implications for disease progression than COPD or asthma alone [3,40,41]. While consensus on the definition of asthma and COPD overlap syndrome is limited, a broad definition of chronic respiratory disease with clinical features of both asthma and COPD is generally agreed [42]. However, other qualifications can include semi-reversible airflow limitation, increased eosinophil counts, a history of asthma, a history of smoking or atopy [42]. Since we could not investigate other clinical features and temporality in the current study, it remains unclear whether the identified association is the result of asthma-COPD overlap syndrome or historical asthma affecting long term lung function leading to the eventual development of COPD.

In this study, several occupational exposures had significant associations with COPD occurrence or severity. A study by Dement et al. reported that certain types of trades such as masons (cement, brick, plaster, etc.), mechanics, and carpenters had higher odds of COPD in older DOE construction workers compared to non-construction workers [43]. This is consistent with the observed association between COPD occurrence and occupational exposure to silica dust in this study. Moreover, silica dust has also been reported to have an association with restrictive lung diseases such as silicosis [44]. Suffice it to say that the findings from the current study are consistent with those from several other studies [8,45,46] that reported associations between silica dust and occurrence of obstructive lung conditions, including COPD. Contrary to reports from other published literature [5,47–49], the current study observed an association between diesel exhaust exposure and higher COPD severity. However, a previous study by Zhang et al. did examine long-term effects of diesel exhaust on lung function and reported a significant association between decreased lung function and diesel exhaust exposure [48]. Since the severity level of COPD is based on grading lung function measurements, specifically the forced expiratory volume in one second (FEV_1_), the results of the current study seem to support those of Zhang et al. [48].

### Strengths and limitations

The use of records from a large nation-wide cohort of over 17,000 DOE former workers in this study allowed for the assessment of individual occupational exposures of interest including silica, diesel exhaust, and welding fumes. In many previous studies assessing occupational exposures and COPD [8,50–52], the exposures of interest were combined into single generic variable of vapors, dusts, or gases. However, the current study assessed each occupational exposure variable separately, which provides for a more refined analysis of the possible associations of these exposures with COPD occurrence and severity.

A limitation of the study is the limited ability to decipher temporality in the identified associations between COPD and its predictors, which is a factor of cross-sectional study designs, and as such, causal inferences cannot be drawn [26]. Additionally, the NSSP relies on self-reported measures for medical histories and occupational exposures which may be subject to bias [53]. This limitation may be particularly applicable to the association of history of asthma and COPD since the study relies on self-reported history of asthma, without clinical confirmation of disease, for the variable definition. For the outcome, however, the study relies on the application of GOLD criteria to clinical spirometry readings for identification of COPD. Although the GOLD criterion is an accepted standard for COPD, it may overestimate the prevalence especially in the elderly as well as underestimate the associations between risk factors and COPD [13]. Additionally, the investigation of air quality for a possible association with COPD prevalence and severity relied upon the annual mean air quality index in each participant’s residential zip code, which may not accurately represent individual exposure [54]. Finally, the study does not incorporate quantitative measures of occupational exposure because this information is not available in the NSSP database. Therefore, residual confounding is possible since only self-reported occupational exposures were used instead of quantitative values (that were unavailable in the dataset). The exposure potential classification scheme for the twelve standardized job titles, initially developed by a Department of Energy (DOE) surveillance program, was based on the perceived potential for exposure to noxious agents based on the job requirements. However, this classification scheme has some level of subjectivity and hence may be prone to mis-classification bias. Finally, the retrospective data used in this study did not contain genetic information nor did the study include information on comorbidities. Hence, genetic factors and comorbidities were not assessed in this study, but they represent a possible extension in future studies. In addition, the generalizability of these results may be affected by the lack of diversity within the study population as the NSSP cohort is primarily non-black males. Future studies will need to assess a more diverse population. Despite these limitations, this study provides valuable information on predictors of COPD occurrence and severity in an occupational cohort and therefore the findings are useful for improving screening guidelines for occupational cohorts.

## Conclusion

This study identified predictors of COPD occurrence and disease severity in the NSSP cohort. In addition to confirming the importance of historically prominent predictors of COPD such as smoking and age, this study demonstrates the importance of considering the occupational experience for both COPD occurrence and severity. This information may be important for occupational screening programs as well as aiding in identifying modifiable risk factors to guide prevention efforts.
